# The Relationship of Urinary Metabolites of Carbaryl/Naphthalene and Chlorpyrifos with Human Semen Quality

**DOI:** 10.1289/ehp.7234

**Published:** 2004-09-07

**Authors:** John D. Meeker, Louise Ryan, Dana B. Barr, Robert F. Herrick, Deborah H. Bennett, Roberto Bravo, Russ Hauser

**Affiliations:** ^1^Department of Environmental Health and; ^2^Department of Biostatistics, Harvard School of Public Health, Boston, Massachusetts, USA; ^3^Centers for Disease Control and Prevention, Atlanta, Georgia, USA

**Keywords:** biological markers, environment, human, pesticides, semen

## Abstract

Most of the general population is exposed to carbaryl and other contemporary-use insecticides at low levels. Studies of laboratory animals, in addition to limited human data, show an association between carbaryl exposure and decreased semen quality. In the present study we explored whether environmental exposures to 1-naphthol (1N), a metabolite of carbaryl and naphthalene, and 3,5,6-trichloro-2-pyridinol (TCPY), a metabolite of chlorpyrifos and chlorpyrifos-methyl, are associated with decreased semen quality in humans. Subjects (*n* = 272) were recruited through a Massachusetts infertility clinic. Individual exposures were measured as spot urinary concentrations of 1N and TCPY adjusted using specific gravity. Semen quality was assessed as sperm concentration, percent motile sperm, and percent sperm with normal morphology, along with sperm motion parameters (straight-line velocity, curvilinear velocity, and linearity). Median TCPY and 1N concentrations were 3.22 and 3.19 μg/L, respectively. For increasing 1N tertiles, adjusted odds ratios (ORs) were significantly elevated for below-reference sperm concentration (OR for low, medium, and high tertiles = 1.0, 4.2, 4.2, respectively; *p*-value for trend = 0.01) and percent motile sperm (1.0, 2.5, 2.4; *p*-value for trend = 0.01). The sperm motion parameter most strongly associated with 1N was straight-line velocity. There were suggestive, borderline-significant associations for TCPY with sperm concentration and motility, whereas sperm morphology was weakly and nonsignificantly associated with both TCPY and 1N. The observed associations between altered semen quality and 1N are consistent with previous studies of carbaryl exposure, although suggestive associations with TCPY are difficult to interpret because human and animal data are currently limited.

Despite the ubiquitous use of insecticides and subsequent exposure among the general population [Centers for Disease Control and Prevention (CDC) 2003; Hill et al. 1995; MacIntosh et al. 1999], there are limited human studies investigating associations between exposure to contemporary-use insecticides at environmental levels and male reproductive health. Human and animal data suggest a potential association between exposures to some commonly used insecticides and decreased semen quality. A study of workers that packaged carbaryl found an increased proportion of oligozoospermic (< 20 million sperm/mL) and teratospermic (> 60% abnormal sperm morphology) men compared with a reference group of chemical workers (Whorton et al. 1979; Wyrobek et al. 1981). Further support for carbaryl’s testicular toxicity comes from studies in laboratory rats that showed associations between carbaryl exposure and sperm shape abnormalities and chromosomal aberrations (Luca and Balan 1987), as well as dose–response relationships between carbaryl exposure and a decline in epididymal sperm count and motility and increased abnormal sperm morphology (Pant et al. 1995, 1996; Rybakova 1966; Shtenberg and Rybakova 1968). Carbaryl was also found to disrupt endocrine regulation of gonadal function in fish (Ghosh and Bhattacharya 1990). Chlorpyrifos, a frequently used insecticide until being banned for residential use (Lewis 2000), is less studied than is carbaryl for its testicular toxicity but has been found to disrupt endocrine regulation in ewes (Rawlings et al. 1998). Recently, the CDC reported measurable levels of urinary 3,5,6-trichloro-2-pyridinol (TCPY), a metabolite of chlorpyrifos and chlorpyrifos-methyl, and 1-naphthol (1N), a metabolite of carbaryl and naphthalene, in > 90% and 75% of males in the United States, respectively (CDC 2003).

The present study was designed to investigate the association between environmental exposure to the nonpersistent insecticides chlorpyrifos and carbaryl and altered semen quality among adult men. Insecticide metabolite levels in urine were used as biomarkers of chlorpyrifos and carbaryl exposure.

## Materials and Methods

Study subjects were men who were partners in subfertile couples seeking infertility diagnosis from the Vincent Burnham Andrology lab at Massachusetts General Hospital (Boston, MA) between January 2000 and April 2003. The study was approved by the human studies institutional review boards of the Massachusetts General Hospital and the Harvard School of Public Health. After the study procedures were explained and all questions answered, subjects signed informed consent forms. Details of subject recruitment have been previously described (Hauser et al. 2003). Briefly, consecutive eligible men were recruited to participate. Of those approached, 65% consented. Most men who declined to participate in the study cited lack of time on the day of their clinic visit as the reason for not participating. Men with a medical history of risk factors for infertility (e.g., varicocele or orchidopexy) were *a priori* excluded from the study analyses. None of the men reported occupational exposure to pesticides or other agents suspected to be associated with semen quality. A single spot urine sample was collected from each subject on the same day as the semen sample. Urine samples were frozen at −20°C and mailed on dry ice to the CDC, where TCPY and 1N were measured as previously described by Hill et al. (1995). Briefly, samples were fortified with stable isotope analogs of the target analytes, and glucuronide or sulfate-bound metabolites were liberated using an enzyme hydrolysis. TCPY and 1N were isolated using liquid–liquid extraction, chemically derivatized, and measured using gas chromatography–chemical ionization–tandem mass spectrometry.

Although creatinine concentrations are commonly used to adjust for variable urine dilution in spot samples when measuring pesticide metabolites, creatinine adjustment may not be appropriate for compounds that undergo active tubular secretion, which includes organic compounds such as TCPY and 1N that can be conjugated by the liver in the form of glucuronides or sulfates (Boeniger et al. 1993). Creatinine levels also vary by sex, age, muscle mass, race, diet, activity, and time of day. Therefore, adjusting urine insecticide metabolite concentrations using specific gravity (SG) may be more appropriate; thus, SG was used as the primary method for dilution adjustment in the present study. However, in addition to SG-adjusted results, volume-based (unadjusted) and creatinine-adjusted TCPY and 1N concentrations were also determined to allow for comparisons with exposure distributions from other studies. Samples with creatinine concentrations > 300 mg/dL or < 30 mg/dL, or with SG > 1.03 or < 1.01, were considered too concentrated or too dilute, respectively, to provide valid results (Teass et al. 1998) and were excluded. Creatinine was measured photo-metrically using kinetic colorimetric assay technology with a Hitachi 911 automated chemistry analyzer (Roche Diagnostics, Indianapolis, IN). SG was measured using a handheld refractometer (National Instrument Company Inc., Baltimore, MD).

Measurement of the semen parameters (sperm concentration, motility, and morphology) has been described previously (Hauser et al. 2003). Briefly, we measured sperm count and motility by computer-aided semen analysis (CASA) using the Hamilton Thorne IVOS 10 Analyzer (Hamilton-Thorne Research, Beverly, MA). To assess sperm morphology, we evaluated 200 sperm using the Tygerberg Strict Criteria (Kruger et al. 1988). In addition, seven CASA motion parameters were measured. Measurement of these parameters has been previously described (Duty et al. 2004). Briefly, CASA outcomes included a mathematically smoothed velocity (designated VAP), straight-line velocity (VSL), curvilinear velocity (VCL), amplitude of lateral head displacement (ALH) that corresponds to the mean width of the head oscillation as the cell swims, and beat cross frequency (BCF), which measures the frequency with which the cell track crosses the cell path in either direction. VAP, VSL, straightness (STR = VSL/VAP × 100), and linearity (LIN = VSL/VCL × 100) are indicators of sperm progression, whereas VCL, ALH, and BCF are indicators of sperm vigor. We also used STR and LIN to describe sperm swimming pattern. Some of the CASA parameters were strongly correlated with each other because they describe different aspects of the same movement. Measures of progression, VAP and VSL, were highly correlated, which indicated they were likely measuring a similar characteristic of sperm movement. We chose VSL over VAP as a measure of progression because it is a direct measurement as opposed to a mathematically smoothed value. VCL was chosen as a measure of vigor and was strongly and positively correlated with ALH but not correlated with BCF. The two measures of swimming pattern (LIN and STR) were strongly correlated, indicating they were likely measuring a similar characteristic of sperm movement. We chose LIN as a measure of swimming pattern because the other parameters chosen for this study (VSL and VCL) are components of LIN and not of STR. Therefore, we chose measure of progression (VSL), vigor (VCL), and swimming pattern (LIN) for statistical analyses. These three measures are also not as heavily dependent on the type of CASA instrument used, allowing for some comparison with results from other studies.

### Statistical analysis.

Statistical analyses were performed using semen parameters both as a continuous measure and dichotomized using World Health Organization (WHO) reference values for sperm concentration (< 20 million sperm/mL) and motility (< 50% motile sperm; WHO 1999). We used the Tygerberg Strict Criteria for morphology to determine below-reference morphology (< 4% normal morphology) (Kruger et al. 1988). Men with values above reference values for all three semen parameters were used as comparison subjects in the logistic regression models. For the CASA motion parameters (VSL, VCL, and LIN), we used multiple linear regression models to assess associations with insecticide metabolites. Nine azoospermic men were excluded from the CASA analyses because motion parameters were not measurable.

Insecticide metabolite concentrations were used both as a continuous measure and categorized into tertiles. For metabolite values below the limit of detection (LOD), corresponding to 0.25 μg/L for TCPY and 0.40 μg/L for 1N, an imputed value equal to one-half the LOD was used. Normality of the metabolite concentrations and semen parameters was assessed, and appropriate transformations were performed before linear regression. Distributions of TCPY, 1N, and sperm concentration were log-transformed in the models. The remaining semen parameters and CASA parameters were normally distributed and not transformed. Semen parameters were stratified by demographic categories to investigate the potential for confounding. Associations between demographic variables and insecticide metabolite levels were also explored. We considered smoking status, race, age, body mass index, a previous exam for infertility, and abstinence time as potential covariates. Inclusion of covariates in the models was based on statistical and biological considerations (Hosmer and Lemeshow 1989). Covariates were entered into the models individually in a forward stepwise manner. Covariates that changed the exposure parameter estimate by greater than 10% were retained in the multivariate model and were considered confounders. There was evidence of confounding by both age and abstinence time in many, but not all, of the models for the various outcome measures. However, because there is evidence that age and abstinence time are associated with semen quality, we included them in all multivariate models (Blackwell and Zaneveld 1992; Kidd et al. 2001). Age was modeled as a continuous independent variable. Abstinence time was modeled as an ordinal variable with five categories: ≤ 2, 3, 4, 5, and ≥ 6 days.

## Results

A total of 330 eligible men provided a single semen and urine sample. The distributions of urinary levels of TCPY and 1N for the 330 men are presented in Table 1, as are adjusted metabolite distributions after excluding men with highly concentrated or dilute samples according to creatinine (23 of 330 men; *n* = 307) or SG (58 of 330 men; *n* = 272). SG-adjusted TCPY and 1N levels were moderately correlated (Spearman correlation coefficient = 0.3; *p* < 0.001). Demographic characteristics and semen parameters are described in Table 2. Subjects were primarily white (82%), with a mean (± SD) age of 36.2 ± 5.5 years, and 72% had never smoked. The proportion of men with a previous exam for infertility was higher among all three of the below-reference semen parameter groups (48%, 36%, and 40% for sperm concentration, motility, and morphology groups, respectively) than among the comparison group (25%). The semen parameter categories were not mutually exclusive. A man could contribute data to one, two, or all three of the below-reference groups.

Odds ratios (ORs) for the relationship between dichotomized semen parameters and SG-adjusted metabolite tertiles are presented in Table 3. Compared with men in the lowest 1N tertile, men in both the medium and high SG-adjusted 1N tertiles were more likely to have below-reference sperm concentration {ORs for increasing exposure tertiles = 1.0, 4.2 [95% confidence interval (CI), 1.4–13.0], 4.2 [95% CI, 1.4–12.6]; *p*-value for trend = 0.01} and sperm motility [1.0, 2.5 (95% CI, 1.3–4.7), 2.4 (95% CI, 1.2–4.5); *p*-value for trend = 0.01]. Although the ORs for the second and third tertiles were both significantly different from 1.0, the exposure–response trends were not monotonic. There were suggestive associations between SG-adjusted TCPY with sperm concentration (1.0, 2.1, 2.4; *p*-value for trend = 0.09) and sperm motility (1.0, 1.6, 1.7; *p*-value for trend = 0.09). However, the estimates for the second and third tertiles suggest that the dose–response relationship was not monotonic. Sperm morphology was weakly associated with both TCPY and 1N.

**Figure 1:**
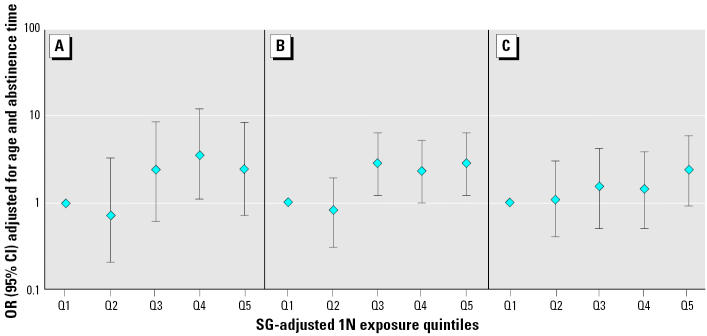
Adjusted ORs and 95% CIs for below-reference semen parameters by increasing quintiles of 1N for (*A*) sperm concentration (*p*-value for trend = 0.02), (*B*) motility (*p*-value for trend = 0.002), and (*C*) morphology (*p*-value for trend = 0.09). The quintiles of SG-adjusted 1N (μg/L) are as follows: Q1 (low), < LOD to 1.50; Q2, 1.50–2.67; Q3, 2.67–3.73; Q4, 3.73–5.86; Q5 (high), 5.86–159.7.

**Figure 2:**
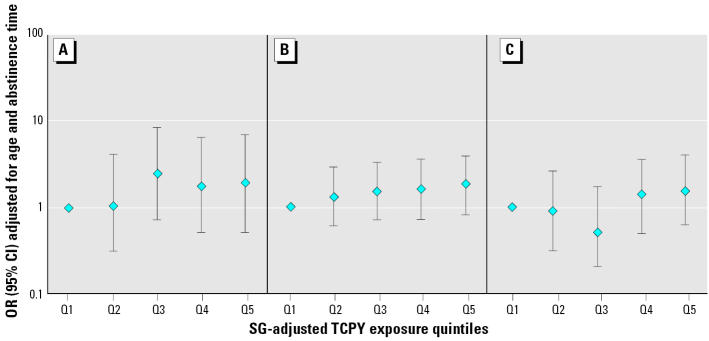
Adjusted ORs and 95% CIs for below-reference semen parameters by increasing quintiles of TCPY for (*A*) sperm concentration (*p*-value for trend = 0.21), (*B*) motility (*p*-value for trend = 0.15), and (*C*) morphology (*p*-value for trend = 0.26). The quintiles of SG-adjusted TCPY (μg/L) are as follows: Q1 (low), < LOD to 1.45; Q2, 1.45–2.72; Q3, 2.72–3.85; Q4, 3.85–5.59; Q5 (high), 5.59–40.69.

To further explore potential dose–response relationships, subjects were divided into quintiles based on SG-adjusted 1N and TCPY concentrations (Figures 1 and 2). Although not monotonic, there were relationships between increased 1N and sperm concentration (OR estimates for increasing exposure quintiles were 1.0, 0.7, 2.3, 3.6, 2.4; *p*-value for trend = 0.02) and decreased sperm motility (1.0, 0.8, 2.8, 2.0, 2.8; *p*-value for trend = 0.002). A suggestive relationship was found between 1N and abnormal sperm morphology (1.0, 1.1, 1.5, 1.4, 2.3; *p*-value for trend = 0.09). Point estimates for the associations between TCPY quintiles and below-reference sperm concentration, motility, and morphology were > 1.0, but none of them approached statistical significance.

We conducted sensitivity analyses to test the robustness of the results. Associations between SG-adjusted exposure tertiles and below-reference semen parameters were recalculated after excluding nine azoospermic men. For 1N, ORs were moderately attenuated for sperm concentration (1.0, 3.0, 3.1; *p*-value for trend = 0.05) but were unchanged for sperm motility. ORs for the highest TCPY tertile with both sperm concentration and motility were slightly larger but remained of borderline statistical significance.

We also reanalyzed the data after retaining the 58 men with SG < 1.01 or > 1.03 (*n* = 330). Estimates of relationships with 1N tertiles became moderately lower for sperm concentration (1.0, 3.0, 2.6; *p*-value for trend = 0.05) and motility (1.0, 2.2, 1.9; *p*-value for trend = 0.03). The suggestive relationship between TCPY tertiles and sperm concentration became slightly stronger (1.0, 1.8, 2.2; *p*-value for trend = 0.08), whereas relationships of 1N with sperm morphology and TCPY with sperm motility and morphology remained weak.

Results of multivariate linear regression models for continuous semen parameters and continuous urinary metabolites are shown in Table 4. A suggestive association between SG-adjusted 1N concentration and decreased sperm concentration was found (*p* = 0.06). As in the logistic regression analysis, there was an association between 1N levels and a decreased percentage of motile sperm (*p* = 0.03). SG-adjusted TCPY did not show associations with decreased concentration or morphology, but there was a suggestive association with motility. Similar results were found in sensitivity analyses that excluded nine azoospermic men (data not shown).

Multivariate linear regression analyses for CASA motion parameters (Table 4) showed significant inverse associations for VSL and LIN with increased SG-adjusted TCPY (*p*-values < 0.05). SG-adjusted 1N levels were inversely associated with VSL (*p* = 0.02). CASA motion parameters were also modeled against tertiles of SG-adjusted TCPY and 1N. The association of TCPY with LIN became nonsignificant (linear regression coefficients for increasing exposure tertiles were 0, −1.16, −1.05; *p*-value for trend = 0.3). An inverse relationship remained for TCPY and VSL (0, −0.13, −2.79; *p*-value for trend = 0.05) and between 1N and VSL (0, −2.17, −3.50; *p*-value for trend = 0.01). There was a suggestive inverse relationship between 1N and VCL (0, −0.49, −4.16; *p*-value for trend = 0.09).

In addition to SG-adjusted values, all statistical analyses were performed with unadjusted and CRE-adjusted TCPY and 1N concentrations (results available from the authors upon request). Results using unadjusted values were similar to those from SG-adjusted values. Creatinine-adjusted results differed from SG-adjusted results. The only relationship in the multivariate logistic models that approached statistical significance was between sperm motility and creatinine-adjusted 1N tertiles (1.0, 1.3, 1.7; *p*-value for trend = 0.08) and quintiles (1.0, 1.3, 1.6, 1.9, 1.8; *p*-value for trend = 0.07). No statistically significant associations were found between creatinine-adjusted metabolite levels and outcome measures in the multivariate linear regression analysis.

## Discussion

In the present study we found associations between urinary metabolites of contemporary-use insecticides and decreased sperm concentration and motility in humans. Specifically, we found statistically significant inverse dose–response relationships between 1N and sperm concentration and motility, as well as between 1N and VSL. Suggestive associations were found between 1N and sperm morphology, VCL, and LIN and between TCPY and sperm concentration, motility, and VSL.

The present data were generally consistent with laboratory animal studies that have shown an association between exposure to carbaryl and decreased semen quality. A 90-day study of rats found statistically significant dose-related declines in epididymal sperm count and percent motile sperm, as well as increased sperm with abnormal morphology (Pant et al. 1995, 1996). In an earlier study, subacute and chronic reproductive effects of carbaryl were found in male rats (Rybakova 1966; Shtenberg and Rybakova 1968). Subacute exposure induced a decrease in motile sperm by an average of 40% after 50 days, whereas chronic exposure led to a significant decrease in motile sperm among even the lowest of the three exposed groups after 12 months.

Limited animal studies have explored relationships between chlorpyrifos exposure and semen quality. Decreased sperm production and motility was observed in Holstein bulls 6 months after dermal lice treatment with an unknown amount of chlorpyrifos [Agency for Substances and Disease Registry (ATSDR) 1997; Everett 1982]. Other animal studies found no associations between chlorpyrifos exposure and altered male reproductive health (ATSDR 1997; Breslin et al. 1996). However, semen quality was not assessed in these studies, and conclusions were reached in part based on the lack of observed changes in testicular weight. In the carbaryl studies, no change in rat testicular weight was reported for lower doses for which decreased semen quality was observed (Pant et al. 1995, 1996; Rybakova 1966).

Human studies investigating exposure to carbaryl and chlorpyrifos and associations with male reproductive health are limited. Until recently, there were no known human male reproductive health studies that used biological measures of exposure to carbaryl and chlorpyrifos (ATSDR 1997). Swan et al. (2003) found elevated but nonsignificant ORs for low semen quality (sperm concentration, motility, and morphology below the population median) among 24 Missouri men with detectable 1N (OR = 2.7; 95% CI, 0.2–34.2) and TCPY levels (6.4; 95% CI, 0.5–86.3). The numbers of subjects were small, limiting statistical power. In a study among Chinese workers exposed to other organophosphate pesticides (ethylparathion and methamidophos), Padungtod et al. (2000) found significantly lower sperm concentration and sperm motility compared with nonexposed workers but no difference in sperm morphology.

In the present study, the relationship between 1N and sperm concentration below the WHO reference value (WHO 1999) is consistent with two published reports on a cohort of carbaryl production workers (Whorton et al. 1979; Wyrobek et al. 1981). Whorton and co-workers found a higher percentage of exposed workers (15%) had sperm concentrations below the reference value of 20 million sperm/mL compared with non-exposed controls (5.5%, *p* = 0.07). In contrast to the present study, Wyrobek et al. (1981) reported an association between carbaryl exposure and sperm morphology. The distribution of abnormal sperm morphology was significantly higher for exposed workers (*p* < 0.005), and the proportion of teratospermic men was larger in the exposed group (29%) compared with controls (12%, *p* = 0.06). Because of logistical constraints, sperm motility was not measured in the published reports of the carbaryl production worker study.

Functional defects of sperm may be an important factor in male infertility. The role of reactive oxygen species (ROS) in male infertility has been suggested in studies that found higher seminal ROS levels in infertile men compared with fertile controls (Agarwal et al. 1994; Pasqualotto et al. 2000). Sperm cells do not have cytoplasmic defense enzymes (e.g., catalase) that serve as ROS scavengers. Consequently, sperm, which have a high content of polyunsaturated fatty acids, are more susceptible to the oxidative deterioration of polyunsaturated fatty acids known as lipid peroxidation (Sharma and Agarwal 1996). Lipid peroxidation causes the plasma membrane to lose its fluidity and integrity, ultimately leading to loss of sperm function (Aitken 1995). Loss of membrane fluidity also impairs the cell membrane ion exchange that controls sperm movement (Rao et al. 1989). Carbaryl causes lipid peroxidation at low concentrations by either efficiently lowering the intracellular level of glutathione, which is associated with an increase in ROS, or through the inhibition of excision esterases (Soderpalm-Berndes and Onfelt 1988). Thus, it is biologically plausible that exposure to carbaryl may be associated with altered semen quality, particularly sperm motility and sperm motion.

Biomonitoring for insecticide metabolite concentrations in urine is a commonly used indicator of internal dose integrating the various routes through which the contaminant enters the body (Barr et al. 1999). However, nonpersistent insecticides are metabolized and excreted rapidly. For example, TCPY has an estimated half-life of 27 hr in humans (Nolan et al. 1984), and levels of both TCPY and 1N measured in urine reflect insecticide exposure in the previous 24–48 hr (Maroni et al. 2000). Spermatogenesis is a cyclical process that takes approximately 3 months. Although insecticide metabolite levels in urine can vary considerably over time, suggesting that a single urine sample may not be a reliable surrogate for longer-term exposure (MacIntosh et al. 1999), we recently showed that a single urine sample was predictive of the 3-month average urinary insecticide metabolite levels (Meeker et al., in press). A single urine sample correctly classified men in the highest 3-month exposure tertile with a sensitivity (specificity) of 0.6 (0.9) for SG-adjusted 1N and 0.5 (0.8) for SG-adjusted TCPY.

Distributions of unadjusted and creatinine-adjusted TCPY and 1N levels in the present study were compared with those recently reported for males in the National Health and Nutrition Examination Survey (NHANES) 1999–2000 (CDC 2003). Unadjusted TCPY concentrations were slightly higher in the present study, with median and 95th percentile values of 2.69 and 10.6 μg/L, respectively, compared with 1.90 and 9.9 μg/L from NHANES 1999–2000. Median and 95th percentiles for unadjusted 1N concentrations were also higher in the present study (2.86 and 13.3 μg/L, respectively, vs. 1.40 and 11.0 μg/L from NHANES 1999–2000). SG-adjusted TCPY and 1N distributions were not reported by NHANES 1999–2000 (CDC 2003).

In the present study, we obtained similar results using SG-adjusted or unadjusted urine metabolite levels, but our results were different for creatinine-adjusted levels. The inability to detect associations using creatinine-adjusted values may reflect tubular secretion of 1N and thus excretion rates of 1N that are independent of urine flow through the glomerulus and not directly related to the amount of creatinine that is filtered (Boeniger et al. 1993). Adjustment of 1N concentration by urinary dilution using creatinine may introduce additional nondifferential exposure measurement error, further limiting the ability to find associations between exposure and outcome.

Strengths of the present study include its size and high participation rate and the use of biological markers of exposure. To test the robustness of the data analysis, we used several modeling approaches in which exposures and outcomes were used as both continuous and categorical measures. The results were consistent across modeling approaches, suggesting that the data were not sensitive to the statistical analysis methods used. Study weaknesses included collecting only a single urine sample as an estimate of 3-month exposure and collecting only a single semen sample to assess semen quality. However, our earlier work supported the utility of a single urine specimen as predictive of 3-month average exposure (Meeker et al., in press). In conclusion, associations between 1N and sperm concentration and motility were found that are consistent with animal studies of carbaryl exposure. The sperm motion parameter most strongly associated with urinary 1N was VSL, although suggestive associations of 1N with VCL and LIN were also found. There were also suggestive associations between TCPY and sperm concentration and motility, but they are difficult to interpret because there are currently limited human and animal data.

Because most of the U.S. population is exposed to these insecticides (CDC 2003), the public health significance of an association with semen quality is potentially large. For instance, our results suggest that an interquartile range increase in carbaryl metabolite levels in urine is associated with a 4% decrease in sperm motility. Although this may not alter an individual man’s fertility, a 4% decrease in the mean of the distribution of sperm motility among U.S. men may result in a significant increase in the number of men in the lower tail of the sperm motility distribution, increasing the number of subfertile men. Further studies are needed to confirm these preliminary findings and assess the potential public health significance.

## Figures and Tables

**Table 1:** Distribution of insecticide (carbaryl and chlorpyrifos) metabolite levels in urine.

Insecticide metabolite	No.*^a^*	Geometric mean	Selected percentiles						
			10th	25th	50th	75th	90th	95th	Maximum
Unadjusted (μg/L)*^b^*									
1N	330	2.82	0.93	1.61	2.86	4.49	7.61	13.28	139.7
TCPY	330	2.32	0.50	1.49	2.69	4.80	7.60	10.57	32.21
SG-adjusted*^c^*									
1N	272	3.13	1.02	1.80	3.19	5.03	9.57	13.96	159.7
TCPY	272	2.63	0.58	1.75	3.22	5.03	7.89	9.66	40.69
CR-adjusted*^d^*									
1N	307	2.32	0.72	1.26	2.21	4.38	7.38	11.04	150.7
TCPY	307	1.97	0.56	1.27	2.29	3.57	5.58	7.08	35.13

a Number of subjects.

b LOD for 1N = 0.40 μg/L; 99.7% of samples > LOD. LOD for TCPY = 0.25 μg/L; 93.9% of samples > LOD.

c Excluded 58 samples with SG > 1.03 or < 1.01.

d Excluded 23 samples with creatinine > 300 or < 30 mg/dL.

**Table 2:** Demographic categories by semen parameters*^a^* (*n* = 330).

	Comparison subjects (*n* = 157)	Sperm concentration < 20 million/mL (*n* = 44)	Sperm motility < 50% motile (*n* = 147)	Sperm morphology < 4% normal (*n* = 72)
Age (mean ± SD)	35.4 ± 5.2	37.6 ± 6.0	37.0 ± 5.6	36.7 ± 5.6
Abstinence time [*n* (%)]				
≤ 2 days	34 (22)	17 (40)	37 (25)	13 (18)
3 days	52 (33)	9 (20)	44 (30)	23 (32)
4 days	28 (18)	6 (14)	24 (16)	12 (17)
5 days	18 (12)	2 (5)	14 (9)	5 (7)
≥ 6 days	24 (15)	9 (20)	27 (18)	19 (26)
Race [*n* (%)]				
White	134 (85)	32 (73)	113 (76)	59 (82)
Black/African American	7 (4)	4 (9)	11 (7)	5 (7)
Hispanic	5 (3)	2 (5)	11 (7)	3 (4)
Other	11 (7)	6 (14)	13 (9)	5 (7)
Smoking status [*n* (%)]				
Never smoker	117 (75)	25 (59)	102 (70)	48 (67)
Ever smoker				
Current smoker	12 (8)	6 (14)	12 (8)	8 (11)
Ex-smoker	27 (17)	11 (25)	30 (20)	15 (21)
Previous exam for infertility [*n* (%)]	40 (25)	21 (48)	54 (36)	29 (40)

a Information on race missing for one man and on smoking for three men.

**Table 3:** Adjusted ORs*^a^* (95% CIs) for SG-adjusted metabolite tertiles (*n* = 272).*^b^*

		Sperm concentration (< 20 million/mL; *n* = 35)		Sperm motility (< 50% motile; *n* = 119)		Sperm morphology (< 4% normal; *n* = 59)	
	Comparison subjects (*n* = 130)	No.*^c^*	OR (95% CI)	No.*^c^*	OR (95% CI)	No.*^c^*	OR (95% CI)
1N*^d^*							
Low	53	5	1.0	27	1.0	20	1.0
Medium	39	14	4.2 (1.4–13.0)*	45	2.5 (1.3–4.7)*	17	1.4 (0.6–3.0)
High	38	16	4.2 (1.4–12.6)*	47	2.4 (1.2–4.5)*	22	1.6 (0.8–3.5)
*p*-Value for trend			0.01		0.01		0.20
TCPY*^e^*							
Low	52	8	1.0	33	1.0	17	1.0
Medium	39	12	2.1 (0.8–5.6)	40	1.6 (0.8–3.0)	16	1.2 (0.5–2.7)
High	39	15	2.4 (0.9–6.3)	46	1.7 (0.9–3.2)	26	1.9 (0.9–4.0)
*p*-Value for trend			0.09		0.09		0.10

a ORs adjusted for age and abstinence time.

b Excluded 58 subjects with SG > 1.03 or < 1.01.

c Number of subjects in each exposure tertile with below-reference semen parameters. The semen parameter categories were not mutually exclusive; a man could contribute data to one, two, or all three of the below-reference groups.

d SG-adjusted 1N tertiles: low, < LOD to 2.36 μg/L; medium, 2.36–4.02 μg/L; high, 4.02–159.7 μg/L.

e SG-adjusted TCPY tertiles: low, < LOD to 2.30 μg/L; medium, 2.30–4.42 μg/L; high, 4.42–40.7 μg/L.

* *p* < 0.05.

**Table 4:** Adjusted regression coefficients^a,b^ for a change in semen parameters and sperm motion parameters associated with an interquartile range (IQR)*^c^* increase in SG-adjusted insecticide metabolite levels (*n* = 272).

	Coefficient (95% CI)	
	1N*^d^*	TCPY*^d^*
Semen parameters		
Concentration*^e^*	0.84 (0.71–1.01)	0.97 (0.83–1.12)
Motility (percent motile)	^−^3.87 (^−^7.28–^−^0.45)*	^−^2.16 (^−^5.05–0.73)
Morphology (percent normal)	^−^0.15 (^−^0.79–0.49)	0.15 (^−^0.39–0.68)
Motion parameters*^f^*		
VSL	^−^1.64 (^−^2.99–^−^0.27)*	^−^1.21 (^−^2.34–^−^0.08)*
VCL	^−^1.98 (^−^4.33,–0.35)	^−^0.53 (^−^2.47–1.42)
LIN	^−^0.79 (^−^1.79–0.22)	^−^1.07 (^−^1.90–^−^0.24)*

a Regression coefficients were adjusted for age and abstinence time.

b Regression coefficients for motility, morphology, and motion parameters represent the change in semen parameter for an IQR change in insecticide metabolite concentration (0, no change in semen parameter for an IQR change in insecticide metabolite concentration; < 0, a decrease in semen parameter for an IQR change in insecticide metabolite concentration; > 0, an increase in semen parameter for an IQR change in insecticide metabolite concentration).

c 1N IQR = 1.80–5.02 μg/L; TCPY IQR = 1.76–5.01 μg/L.

d 1N and TCPY were log transformed for regression analysis.

e Sperm concentration was log transformed. The coefficient represents a multiplicative change in sperm concentration per IQR change in TCPY or 1N (1.0, no change in sperm concentration for an IQR change in insecticide metabolite concentration; < 1.0, a multiplicative decrease in sperm concentration for an IQR change in insecticide metabolite concentration; > 1.0, a multiplicative increase in sperm concentration for an IQR change in insecticide metabolite concentration).

f VSL, VCL, and LIN analyses not performed on 9 azoospermic men; *n* = 263. TCPY IQR = 1.76–5.08 μg/L; 1N IQR = 1.77–5.02 μg/L.

* *p* < 0.05.
